# Exploring the effect of prolonged fasting on kynurenine pathway metabolites and stress markers in healthy male individuals

**DOI:** 10.1038/s41430-024-01451-7

**Published:** 2024-05-24

**Authors:** Varvara Louvrou, Rima Solianik, Marius Brazaitis, Sophie Erhardt

**Affiliations:** 1 Institute of Sport Science and Innovations, Lithuanian Sports University, Kaunas, Lithuania; 2 Department of Physiology and Pharmacology, Karolinska Institutet, Stockholm, Sweden

**Keywords:** Metabolism, Nutrition

## Abstract

**Background/objectives:**

Prolonged fasting triggers a stress response within the human body. Our objective was to investigate the impact of prolonged fasting, in conjunction with stress, on kynurenine pathway metabolites.

**Subjects/methods:**

Healthy males were divided into fasting group (zero-calorie-restriction) for 6 days (FAST, *n* = 14), and control group (CON, *n* = 10). Blood and saliva samples were collected at baseline, Day 2, Day 4, Day 6 during fasting period, and 1 week after resuming regular diet. Plasma levels of kynurenine pathway metabolites were measured using ultra-performance liquid chromatography-mass spectrometry (UPLC-MS/MS). Plasma and salivary samples were analyzed for stress markers.

**Results:**

A pronounced activation of the kynurenine pathway in individuals on FAST trial was revealed. Concentrations of picolinic acid (PIC), kynurenic acid (KYNA) and 3-hydroxykynurenine (3-HK) were significantly increased, with peak levels observed on Day 6 (*P* < 0.0001). Conversely, concentrations of tryptophan (TRP) and quinolinic acid (QUIN) decreased (*P* < 0.0001), while kynurenine (KYN) and nicotinamide (NAM) levels remained stable. Cortisol and noradrenaline concentrations remained unchanged. However, adrenaline levels significantly increased on Day 4 within FAST compared to CON (*P* = 0.005). Notably, all deviations in kynurenine pathway metabolite levels returned to baseline values upon resuming regular diet following the 6-day fasting regimen, even when weight and BMI parameters were not restored.

**Conclusions:**

Extended fasting over 6 days induces the kynurenine pathway and has minimal effects on stress markers. Restoration of metabolite concentrations upon regular feeding implies rapid adaptation of the kynurenine pathway synthetic enzymes to maintain homeostasis when faced with perturbations.

## Introduction

Fasting is a practice that involves the intentional restriction or complete avoidance of food and caloric beverages for varying durations, spanning from hours to weeks. It is established that fasting induces ketogenesis, leading to significant alterations in metabolic pathways and cellular processes, including stress resistance, lipolysis, and autophagy. Fasting has been consistently highlighted for its dual role in enhancing overall health and serving as a potential disease modifier (see review: [1]). Moreover, it can complement pharmacological interventions, especially in specific conditions like epilepsy and diabetes [2, 3]. Apart from effects on lipid, protein and glucose metabolism, fasting also elicits effects on psychological health, particularly in relation to mood regulation [4, 5]. During fasting, hepatic gluconeogenesis stands out as a pivotal process [4], leading to the production of ketones, serving as vital energy source for the brain. Emerging evidence strongly suggests that the induction of peroxisome proliferator-activated receptor gamma coactivator 1-alpha (PGC-1α) expression in the liver plays a crucial role in regulating energy utilization by boosting ATP production. This is particularly significant during fasting, where severe caloric restriction is imposed [6]. Fasting strongly induces liver expression of PGC-1α, which in turn induces the expression of kynurenine aminotransferase (KAT) enzymes and 2-amino-3-carboxymuconate-6-semialdehyde decarboxylase (ACMSD) [7, 8]. Interestingly, KAT and ACMSD are enzymes in the kynurenine pathway of TRP degradation through which several neuroactive metabolites are produced. Furthermore, it is shown that the kynurenine pathway, via KYN and KYNA agonist activity on the aryl hydrocarbon receptor, plays a role in T cell differentiation [9], consequently impacting immune system activity regarding tolerance and inflammation [10]. Interestingly, a recent study from our group showed that 2 days of fasting decreased TRP and increased plasma levels of the kynurenine pathway metabolites KYNA, 3-HK, PIC and the PIC/QUIN ratio in women [11].

Extended fasting induces a stress response in the human body. The physiology of stress response has two components: activation of the sympathetic-adreno-medullar axis, which acts fast, and the activation of the hypothalamic-pituitary-adrenal axis that acts slowly [12]. The former provides rapid physiological adaptation via secretion of catecholamines, noradrenaline and adrenaline [12]. The latter regulates feedback inhibition loops that involve the pituitary and adrenal glands, which control glucocorticoid production and cortisol release respectively. Cortisol exerts widespread effects due to the extensive distribution of its respective receptors in the body [12]. Importantly, many studies have shown that the kynurenine pathway can be influenced by different stressors, and induced by cortisol [13]. Our hypothesis posits that extended fasting will initiate a stress response, concurrently impacting kynurenine pathway metabolites levels. In this study, we aim to explore the effects of a 6-day fasting regimen on peripheral stress marker levels and plasma concentrations of kynurenine pathway metabolites in a male cohort.

## Materials and methods

### Participants

Twenty-nine subjects were assessed for eligibility. The inclusion criteria were (i) male; (ii) aged between 18 and 44 years; (iii) with body mass index (BMI) from 19.5–29.9 kg/m^2^; (iv) no participation in excessive regular moderate or vigorous physical activity (i.e. 3 times per week) and ≤ 150 min of moderate intensity or ≥ 75 min of vigorous-intensity activity per week; (v) no history of any eating, metabolic, skeletal, cardiovascular, oncological, neuromuscular, mental disabilities or conditions that could be negatively affected by fasting and affect experimental variables; (vi) no history of alcohol dependence or psychotropic drugs dependence and (vii) no blood/needle phobia. Participants were excluded if they were smokers, on any medications or participated in weight reduction programs and/or low-carbohydrate diets. Altogether, twenty-four males met the criteria and agreed to participate. Experiments were performed at the Institute of Sports Science and Innovations, Lithuanian Sports University, from February 2021 to September 2022. Data on 13 out of 14 FAST participants’ glucose, ketone, adrenaline and noradrenaline concentrations for timepoints ‘Baseline’ and ‘Day 6’ have been previously published.

### Experimental protocol and study design

**Fig. 1:**
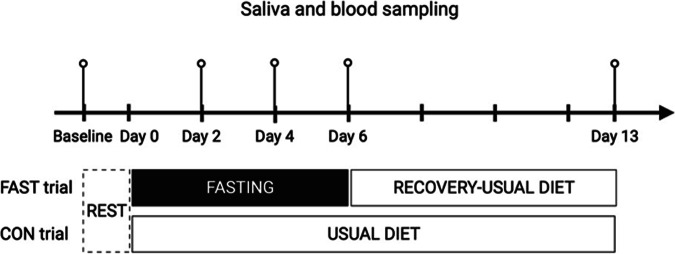
Schematic representation of the experimental protocol for the FAST trial and CON trial. Blood and saliva samples taken at baseline, Day 2, Day 4, Day 6 and Day 13. FAST; fasting (0 kcal/day), CON; usual diet. Created with BioRender.com.

Baseline assessment commenced at 08.00–09.00 h when the participants arrived at the laboratory following an 8–13 h overnight fast. Participants were required to refrain from fatigue-related activities and abstain from ingesting caffeine, medication, and alcoholic beverages for at least 72 h before each experiment assessment. Anthropometric measurements were performed, and the participant rested in a semi-recumbent position for 20 min in a quiet room (temperature 24 °C, 60% humidity). Baseline values of capillary ketone and glucose concentrations were measured, and saliva and venous blood samples were collected (Fig. 1). Participants rested for a day before starting on one of the randomly prescribed trials: 6-day FAST trial (*n* = 14) or CON trial (*n* = 10) (Fig. 1). IBM Statistical Package for the Social Sciences (SPSS) for Windows version 22.0 (IBM Corp., Armonk, NY, USA) was used for randomization. FAST participants were instructed to follow a prescribed zero-calorie diet with water ad libitum over 6 days. CON participants were instructed to maintain their previous eating habits. On the 2nd, 4th, and 6th day of each trial, capillary ketone and glucose concentrations were measured, and saliva and venous blood samples were obtained (Fig. 1). Following a usual-diet period of 7 days (Day 13) after FAST and CON, capillary ketone and glucose concentrations were measured, saliva and venous blood samples were collected.

### Capillary blood ketone and glucose concentrations

The capillary blood ketone and glucose concentrations were assessed via finger-prick capillary test using the Abbott FreeStyle Optium Neo H blood glucose and ketone monitoring system (Doncaster, Australia).

### Blood sample preparation

Venous blood samples from the median antecubital vein were collected into 3 ml vacutainer tubes using EDTA with tri-potassium as an anticoagulant (K3EDTA tube; Fisher Scientific, Waltham, Massachusetts, USA), inverted 8–10 times and kept at 2–8 °C until centrifugation. Plasma was separated by centrifugation at 1200 × g for 15 min at 4 °C within 30 min of blood collection and stored in 0.5 ml aliquots at –80 °C until analysis.

### Plasma preparation for UPLC-MS/MS

Thirty μl of human plasma sample, quality control or standard mix, was mixed with 30 μl of internal standard 0.5 μM in 10% ammonia for 15 s. Next, 60 μl of 200 nM ZnSO4 (5 °C) was added and mixed for 15 s. Then, 30 μl of methanol was added (5 °C) and mixed for 15 s. Additionally, the mixture was centrifuged for 10 min at 2841 × g at room temperature. 30 μl of the supernatant was mixed with 30 μl of 5% formic acid in LC-MS Certified Clear Glass 12 × 32-mm vials (product no. 186005662CV, Waters, Milford, Massachusetts, USA). Samples were transferred to an autosampler (5 °C). The volume injected into the system was 1.5 μl.

### Detection of TRP and KYN metabolites in plasma

Blinded assessors used an UPLC–MS/MS system to measure plasma levels of TRP, KYN, KYNA, 3-HK, PIC, QUIN and NAM. The UPLC–MS/MS system used a Xevo TQ–XS triple quadrupole mass spectrometer (Waters) with a Z-spray electrospray interface and was operated in electrospray positive multiple reaction monitoring mode. UPLC conditions were as follows: separation was carried out using an Acquity UPLC® HSS T3 column (1.8 m, 2.1150 mm, Waters, part number: 186003540) at 50 °C with a guard column (Waters, Vanguard HSS T3 1.8 m, 2.150 mm column, part number: 186003976) to retain impurities from the mobile phase. The mobile phase consisted of: 0.6% formic acid in water (UPLC grade) and 0.6% formic acid in methanol (UPLC grade). The flow rate was 0.3 ml/min. While the MS was operating at a source temperature of 150 °C, the capillary voltage was set to +3.0 kV. The cone gas flow was 150 l/h and the desolvation gas flow rate was 1000 l/h, while the desolvation temperature was 650 °C. The autosampler was set at 5 °C and each sample took 13.0 min to run. All metabolites measured were detected in higher concentrations than the lowest level of quantification (TRP, 10 nM; KYN, 10 nM; KYNA, 10 nM; 3-HK, 10 nM; PIC, 10 nM; QUIN, 50 nM; and NAM, 10 nM). The coefficient of variation (CV) for quality controls (intra-assay, during 15 h) was less than 6% for all metabolites measured. Data processing and acquisition were performed using the software package MassLynx v 4.1 SCN943 SCN979 (© 2016 Waters Inc.). The detailed description of the method has been previously published [14].

### Plasma stress hormones and salivary free cortisol concentrations

The researchers who analyzed saliva and venous blood samples were blinded to the experimental conditions. A minimum of 1.0 ml of saliva samples were collected in microtubes (1.5 ml; FLmedical, Italy) and stored at – 20 °C for later analysis. Cortisol and catecholamines concentrations were measured in duplicates using enzyme-linked immunosorbent assay kits (plasma cortisol: Cat. No. RE52061, catecholamines: Cat. No. RE59242, salivary free cortisol: Cat. No. RE52611, IBL International GmbH, Hamburg, Germany) and a Spark multimode microplate reader (Tecan, Grödig, Austria). The intra-assay CVs were 1.76%, 6.01% and 4.78%, and the inter-assay CVs were 2.42%, 7.32% and 4.94% for plasma cortisol, adrenaline, and noradrenaline, respectively. The intra-assay CV for salivary free cortisol was 3.10% and the inter-assay CV was 9.42%.

### Statistical analysis

An a-priori power analysis was conducted using G*Power version 3.1.9.7 (Düsseldorf, Germany), using data from three participants in each group who completed the study. With a significance criterion of α = 0.05 and power = 0.80, the minimum required sample size was 8 participants per group to detect a main moderate effect of 0.6 for essential amino acid TRP. Furthermore, eight participants (6 in experimental group and 2 in control group) were added after considering the attrition rate and missing data.

Statistical analyses were performed using R programming language (RStudio version 2022.12.0 + 353, RStudio Team, Boston, MA, USA). Unpaired, two-tailed Welch *t*-tests were performed for comparisons between groups using the ggpubr R package. Homogeneity of variance was assessed using the Levene’s test, and all kynurenine pathway metabolite data were log10 transformed before fitting the model. In the case of ketones, the Levene’s test was not passed so robust standard errors were employed before fitting the model. A random linear mixed model with Bonferroni correction test was performed using the lme4 and emmeans R package. Age effect was adjusted as a co-variate factor. Subject was added as a random effect in the model. Pairwise comparisons were used to examine the effect of time within the group and to assess the effect of fasting between groups (FAST vs. CON). Normal distribution of the model residuals was assessed visually with the aid of QQ plots and histograms. All graphs were created using GraphPad Prism 9 (version 9.5.0 (525) GraphPad Software, San Diego, California, USA). Statistical significance was set at *P* < 0.05. Data are presented as the mean and standard error of the mean (SEM).

## Results

At baseline, the groups differed significantly in mean age (FAST 32.1 ± 1.9 vs. CON 23.4 ± 1.0, *P* = 0.0004) and BMI (kg/m^2^) (FAST 25.7 ± 0.7 vs. CON 23.6 ± 0.7, *P* = 0.02), but not in weight (kg) (FAST 88.3 ± 3.9 vs. CON 78.9 ± 4.7, *P* = 0.15). Age is regarded as a co-variate, therefore the data have been adjusted accordingly (Supplementary Table 1).

**Fig. 2:**
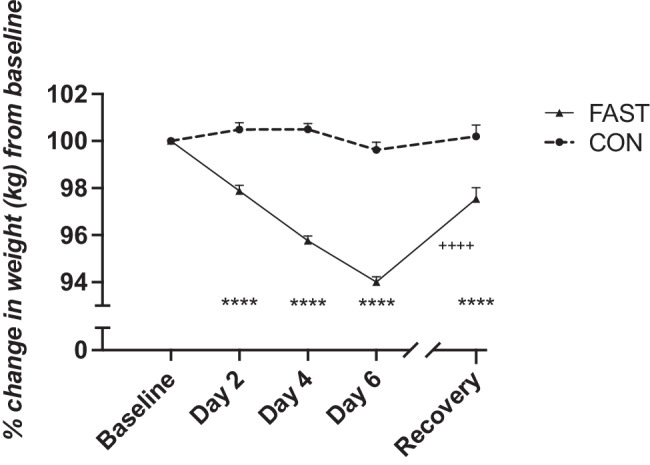
Weight data for FAST and CON groups. Weight was recorded every day during fasting and subsequently 1 week of recovery. Data are shown in mean ± SEM. ****P < 0.0001, compared with FAST group baseline values, ^++++^*P* < 0.0001, for Day 6 -Recovery comparisons within FAST group. FAST; fasting (0 kcal/day), CON usual diet.

Weight experienced a significant decrease during the fasting period (*P* < 0.0001) and did not rebound to baseline values one week after resuming the regular diet (*P* < 0.0001) (Fig. 2). Compliance with fasting regime was supported by capillary glucose and ketone changes. In the FAST trial, glucose concentrations were decreasing steadily (*P* < 0.0001) while ketone concentrations showed a marked increase (*P* < 0.0001). Both analytes returned to baseline values after recovery (Supplementary Fig. 1).

**Fig. 3:**
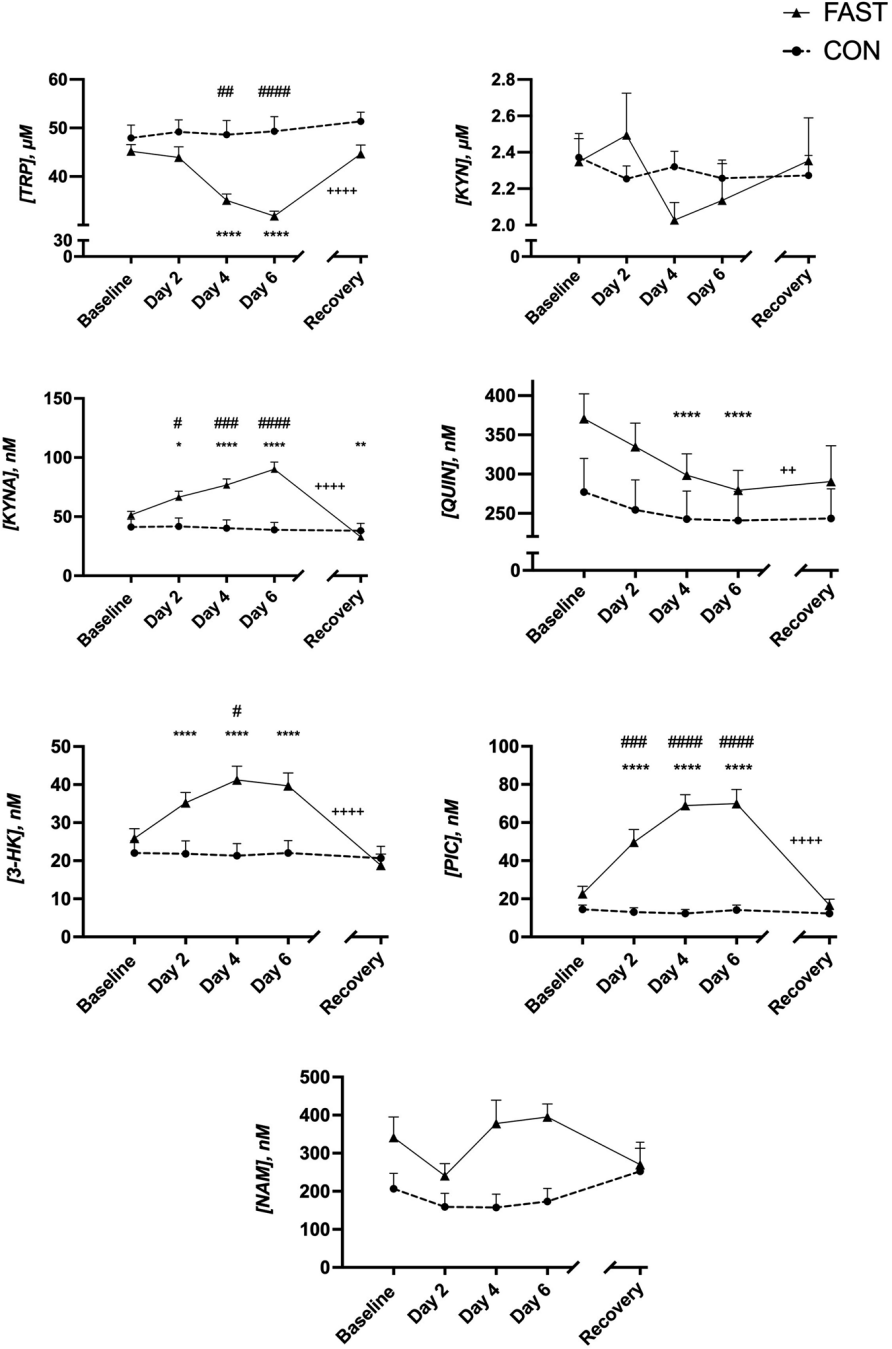
Effects of FAST and CON on kynurenine pathway metabolite concentrations. Data are shown in mean ± SEM. *P < 0.05, **P < 0.01, ****P < 0.0001, compared with FAST baseline values. ^#^*P* < 0.05, ^##^*P* < 0.01, ^###^*P* < 0.001, ^####^*P* < 0.0001 for between group comparisons, ^++^*P* < 0.01, ^++++^*P* < 0.0001, for Day 6 - Recovery comparisons within FAST group. FAST; fasting (0 kcal/day), CON usual diet.

The effect of 6-day fasting on the kynurenine pathway was examined by measuring plasma concentrations of the metabolites (TRP, KYN, KYNA, QUIN, 3-HK, PIC, and NAM), as summarized in Fig. 3. In the CON group, no significant time effects were observed for any of the metabolites (all *P* > 0.05). In contrast, in the FAST group, significant time effects were observed for plasma levels of all metabolites except KYN and NAM. Significant differences were revealed between FAST and CON groups regarding the concentrations of TRP (*P* < 0.0001), KYNA (*P* < 0.0001), 3-HK (*P* < 0.05) and PIC (*P* < 0.0001) during the 6 days of fasting. While concentrations of TRP significantly decreased (*P* < 0.001), concentrations of KYNA, 3-HK and PIC increased significantly, peaking at 6 days of fasting. Although QUIN concentrations decreased significantly in the FAST group (*P* < 0.0001), there were no significant differences between the two groups. All baseline metabolite concentrations were not significantly different from after-recovery metabolite concentrations (*P* > 0.05), except KYNA (*P* = 0.006). For 3-HK, KYNA, and PIC a trend of continuously increasing metabolite concentrations persisted until the sixth day of fasting, followed by a subsequent return to baseline metabolite concentrations after resuming to regular diet.

**Fig. 4:**
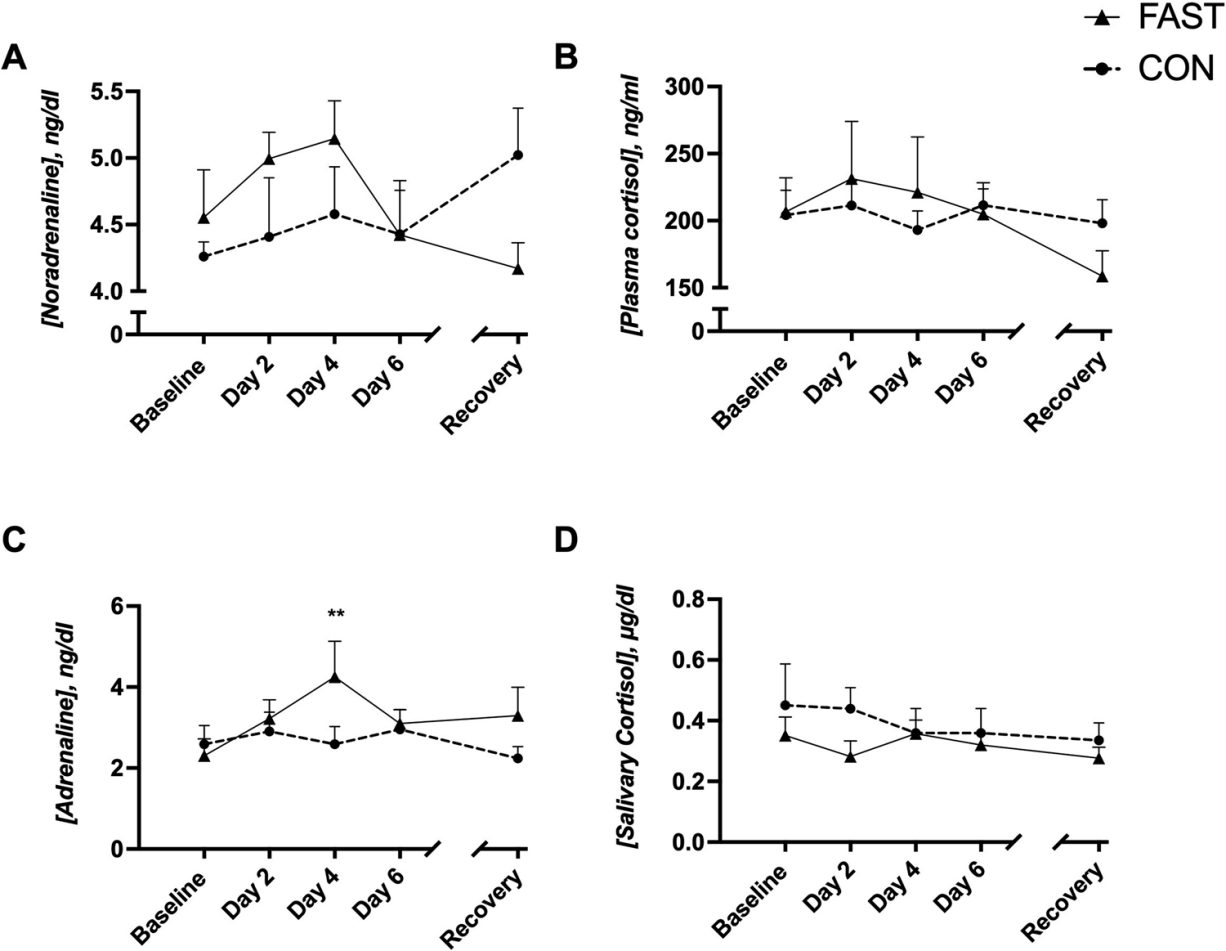
Effects of FAST and CON on stress marker concentrations: salivary and plasma cortisol, noradrenaline, and adrenaline. Data are shown in mean ± SEM. **P < 0.01, compared with FAST baseline values. FAST fasting (0 kcal/day), CON usual diet.

Salivary and plasma cortisol concentrations showed no significant time or group effects (Fig. 4B, D). Although noradrenaline concentrations appear more variable, no significant effects were observed (Fig. 4A). Adrenaline levels showed no significant time effects within CON group but peaked at day 4 for the individuals in FAST group, resulting in a significant increase from baseline FAST value (Fig. 4C, *P* = 0.005).

## Discussion

The kynurenine pathway is the main route for TRP degradation, generating metabolites with widespread functions. The initial step involves conversion of TRP to KYN, catalyzed by enzymes tryptophan 2,3-dioxygenase (TDO) and enzyme indoleamine 2,3-dioxygenase (IDO) [15, 16]. This pathway is implicated in numerous diseases related to immune response and excitatory neurotransmission, inevitably connecting it to neurological and psychiatric disorders [17–19]. It is also associated with malaria, diabetes, gastrointestinal disorders, and cancer [20–22]. KYN, KYNA and QUIN are proposed biomarkers for disease progression and severity [23, 24], emphasizing the necessity of studies on pathway regulation in healthy individuals.

Accumulating evidence from human and animal studies underscores the preventive benefits of fasting against metabolic and inflammatory disorders [25]. Lifestyle changes, like physical activity and dietary restriction, rather than pharmaceuticals, have historically been the primary approach toward therapy.

The present study explores the impact of prolonged fasting, (over 6 days) on stress markers and plasma concentrations of kynurenine pathway metabolites in healthy males. The results reveal activation of the pathway during fasting, where some metabolites show significant increases, while others remain relatively stable. TRP and QUIN plasma levels decreased. Importantly, all metabolite concentrations reverted to their baseline upon resuming a regular diet, except QUIN, which remained below its baseline for unknown reasons. Notably, the metabolites’ return to baseline was not coupled with full recovery of weight and BMI parameters.

TRP concentration was expectedly decreased as it is an essential amino acid obtained from diet. TRP is vital for energy metabolism, contributing to NAD+ and acetyl-CoA formation, used for ATP generation [26]. Principally, TDO enzyme in the liver controls TRP to KYN conversion [16]. TDO activity has been described to be largely regulated by TRP availability but is also influenced by hormones, such as cortisol and insulin [27]. Notably, glucose inhibits TDO activity in rats [28], and our present study reveals a considerable decrease in glucose concentrations during fasting. KYN is the substrate for all subsequent metabolites in the pathway and may be tightly regulated, thus remained constant during fasting. Hence, the heightened activity of TDO may contribute to the elevation of downstream metabolites (KYNA, 3-HK, PIC).

Insulin suppresses the aminocarboxymuconate-semialdehyde decarboxylase (ACSMD) enzyme, inhibiting PIC production [29]. Reduced glucose and insulin levels attributed to fasting [30], alleviate the inhibition of TDO and ACMSD enzymes, aligning with the study’s findings.

Cortisol induces TDO activity, enhancing breakdown of TRP to KYN [31]. Moreover, cortisol concentration significantly increases after 8 or 10 days of water-only fasting in males and females [32, 33].

Chronic stress activates the kynurenine pathway [34, 35], an effect reversed by IDO inhibition [36]. Aerobic exercise may be protective against stress-induced depression via upregulated expression of KAT enzymes in muscle, consequently increasing KYN to KYNA conversion peripherally, which prevents KYN accumulation in the brain [37]. Stressful events also correlate with increased serum 3-HK levels [38].

Contrary to expectations, stress markers, like cortisol and noradrenaline did not significantly differ throughout fasting. Adrenaline levels were significantly increased after 4 days of fasting as compared to baseline, which may reflect sympathetic adreno-medullar axis activation. Although noradrenaline levels followed the same trend and peaked after 4 days of fasting, the change from baseline was insignificant. Due to cortisol circadian rhythm [39], one may speculate that the once a-day measurement of cortisol in the present study was insufficient to reveal a difference between FAST and CON groups cortisol levels throughout the day. In line with this, a study in primates [40] that employed a continuous 9-h cortisol measurement shows that change in cortisol levels after stress is more noticeable later in the day.

Fasting increased plasma concentrations of KYNA, 3-HK and PIC, whereas QUIN decreased significantly as compared to CON. Remarkably, the same shift was described in a previous 2-day fasting study [11], further supporting present findings. Increased PIC concentration might be attributed to PGC-1α involvement, a transcription coactivator that induces ACMSD expression. Indeed, fasting induces ACMSD via glucagon and glucocorticoid signaling [4, 7, 29]. ACMSD can be conceptualized as a regulator influencing the QUIN/PIC balance, favoring an increased PIC concentration and a decreased QUIN concentration in this context. The heightened PIC concentration also signifies an enhanced acetyl CoA synthesis, which enters the tricarboxylic acid cycle generating energy. Mice studies show the energy modulator PGC-1α increasing KAT enzyme expression in skeletal muscle, thus facilitating KYN to KYNA conversion, thereby enhancing energy efficiency and preventing fatigue [8]. Skeletal muscle mitochondria are essential in energy turnover suggesting PGC-1α expression as a critical factor in calorie restriction, energy storage mobilization, and energy propagation [41].

Increased plasma 3-HK concentration may be the product of increased kynurenine 3-monooxygenase enzyme activity. The enzyme can be influenced by anti-inflammatory cytokines such as IL-4 and IL-10, reflecting an anti-inflammatory function of fasting [42]. This aligns with previously published evidence that suggests the anti-inflammatory actions of PGC-1α on PPAR-α/PPAR-γ, as well as B-hydroxybutyrate on NLRP3 inflammasome [43, 44].

In the present study, but also in a recent study [45], we uncovered a reduction in glucose levels and a significant increase in ketone levels after a 6-day fasting period in males. These effects may be attributed to depletion of liver glycogen stores, leading to fatty acid generation, which are subsequently converted into ketones, including acetoacetate and β-hydroxybutyrate. Ketones serve as primary alternative energy source in the brain. Notably, β-hydroxybutyrate has been shown to elevate KYNA levels in brain cortical slices, primary glial cultures, and in rat brain tissues. [46, 47].

Further, a metabolomics study has demonstrated that fasting for 58 h increases ketone, leucine, and nicotinamide levels [48]. Excess leucine inhibits the quinolinate phosphoribosyltransferase enzyme and induces ACMSD, resulting in increased PIC levels [49], which supports this study’s findings.

Finally, it is crucial to evaluate the results’ reproducibility. By comparing the current data at the 2-day fasting timepoint with findings from another study from our lab involving a 2-day fasting regimen in women, we observe highly comparable levels of all metabolites. The persistent consistency in patterns and concentrations across studies reinforces our results [11].

### Limitations

Considering the known sex differences in kynurenine pathway metabolite levels, even under baseline conditions [50], and recognizing that menstrual cycle transitions could significantly influence sex hormone levels, the present study only included males. As a future perspective, a separate study in females, accounting for all three menstrual cycle phases would provide more comprehensive understanding of how metabolic processes vary in females’ hormonal states.

Notably, the FAST and CON group had significantly different mean BMI at baseline. BMI can be considered as a covariate but given the study’s nature, BMI was not adjusted for. Collecting additional blood or saliva samples, especially considering cortisol’s circadian rhythm, would have provided valuable insights. Furthermore, this study did not account for protein binding of the metabolites. Measurements of free metabolites and albumin levels would offer deeper insight into tryptophan metabolism and should be prioritized for future investigations [51, 52].

## Conclusion

To conclude, this study demonstrates the effects of prolonged fasting on kynurenine pathway metabolites in healthy, young males. Recovery of metabolite concentrations to baseline after resuming regular diet, alongside non-restored weight and BMI parameters, suggests a rapid adaptation of kynurenine pathway synthetic enzymes to homeostatic perturbations. Further studies are needed to interpret the broader relevance of the observed fasting metabolic profile to diseases associated with the kynurenine pathway.

### Supplementary information

Supplementary Material

## Data Availability

The data analyzed in the current study are not publicly available due to data confidentiality reasons. Data analyzed in this study are available from the corresponding author upon reasonable request.

## Code availability

We have followed best practices in open science by making all RStudio analysis scripts used in the study openly available on Github (https://github.com/varlouv/6-day-fasting-study.git).
