# Supplementary material for: Exploring the effect of prolonged fasting on kynurenine pathway metabolites and stress markers in healthy male individuals
Source: Eur J Clin Nutr. 2024 May 24;78(8):677–83. doi: 10.1038/s41430-024-01451-7 (PMC11300305; doi:10.1038/s41430-024-01451-7)
Supplement: Supplementary file 1 — Supplementary Material [file 41430_2024_1451_MOESM1_ESM.docx]

**Supplementary Table 1.** Anthropometric data of the participants in the study.

| **Participants** | **Mean age (yr) ± SEM** | **Mean weight (kg) ± SEM** | **Mean BMI (kg/m^2^)**  **± SEM** |
| --- | --- | --- | --- |
| FAST group  (n=14) | 32.1 ± 1.9 | 88.3 ± 3.9 | 25.7 ± 0.7 |
| CON group  (n=10) | 23.4 ± 1.0 | 78.9 ± 4.7 | 23.6 ± 0.7 |

**Supplementary Figure 1.** Glucose and ketone concentrations changes in FAST and CON trials.

Data are shown in mean ± SEM. ***P <0.001, ****P < 0.0001 for comparisons with FAST baseline values. #P < 0.05, ###P < 0.001, ####P < 0.0001, for between group comparisons.
